# Effect of omega-3 polyunsaturated fatty acids on the cytoskeleton: an open-label intervention study

**DOI:** 10.1186/1476-511X-14-4

**Published:** 2015-02-14

**Authors:** Simone Schmidt, Janina Willers, Sabine Riecker, Katharina Möller, Jan Philipp Schuchardt, Andreas Hahn

**Affiliations:** Institute of Food Science and Human Nutrition, Leibniz University of Hannover, Am Kleinen Felde 30, 30167 Hannover, Germany

**Keywords:** n-3 PUFA, Omega-3 index, Cytoskeleton, Gene-expression, F-actin, GTPases, Red blood cells

## Abstract

**Background:**

Omega-3 polyunsaturated fatty acids (n-3 PUFAs) show beneficial effects on cardiovascular health and cognitive functions, but the underlying molecular mechanisms are not completely understood. Because of the fact that cytoskeleton dynamics affect almost every cellular process, the regulation of cytoskeletal dynamics could be a new pathway by which n-3 PUFAs exert their effects on cellular level.

**Methods:**

A 12-week open-label intervention study with 12 healthy men was conducted to determine the effects of 2.7 g/d n-3 PUFA on changes in mRNA expression of cytoskeleton-associated genes by quantitative real-time PCR in whole blood. Furthermore, the actin content in red blood cells was analyzed by immunofluorescence imaging.

**Results:**

N-3 PUFA supplementation resulted in a significant down-regulation of cytoskeleton-associated genes, in particular three GTPases (RAC1, RHOA, CDC42), three kinases (ROCK1, PAK2, LIMK), two Wiskott-Aldrich syndrome proteins (WASL, WASF2) as well as actin related protein 2/3 complex (ARPC2, ARPC3) and cofilin (CFL1). Variability in F-actin content between subjects was high; reduced actin content was only reduced within group evaluation.

**Conclusions:**

Reduced cytoskeleton-associated gene expression after n-3 PUFA supplementation suggests that regulation of cytoskeleton dynamics might be an additional way by which n-3 PUFAs exert their cellular effects. Concerning F-actin, this analysis did not reveal unmistakable results impeding a generalized conclusion.

**Electronic supplementary material:**

The online version of this article (doi:10.1186/1476-511X-14-4) contains supplementary material, which is available to authorized users.

## Background

Fish oil and its principal omega-3 polyunsaturated fatty acids (n-3 PUFAs), eicosapentaenoic acid (EPA) and docosahexaenoic acid (DHA), have shown beneficial effects in the prevention of atherosclerosis and cardiovascular diseases [1, 2]. They are incorporated into cell membranes and show potent anti-inflammatory, anti-thrombotic, anti-arrhythmic, and triacylglycerol-lowering activities [3–5]. In addition, indications for the neuroprotective functions of n-3 PUFAs have been constantly increased in the last few years, including effects on learning memory and prevention of neurodegenerative diseases by the deceleration of cognitive decline [6, 7]. However, the underlying molecular mechanisms by which n-3 PUFAs exert their effects are versatile and not completely understood. The modulation of membrane micro-organization and the regulation of gene expression are believed to be key mechanisms by which n-3 PUFAs mediate their functions [8]. The regulation of gene expression via nuclear receptors and transcription factors enables n-3 PUFAs and their bioactive metabolites to affect a myriad of molecular pathways. In a recent study investigating the effect of n-3 PUFA on the whole genome expression in humans [9–11], we observed significant changes in the expression of several cytoskeleton-associated genes [GSE34898] after a twelve week supplementation with fish oil (1.56 g EPA and 1.14 g DHA) or placebo (corn oil) in n = 20 normolipidemic (see Additional file 1: Table S1) and in n = 20 dyslipidemic subjects (see Additional file 1: Table S2). Independent of the oil, a significantly higher number of genes was regulated in dyslipidemic subjects compared to normolipidemic subjects. Gene lists show regulated genes coding for structural components of the cytoskeleton (actinin, moesin), muscles (dystrophin, myosin) as well as for several factors which are responsible for the regulation of cytoskeleton dynamics (Rho GTPase, WAVE-2), cell shape changes (syndecan) and actin remodeling (Arp 2/3 complex, CapZ beta, WASP). These regulations could be another pathway by which n-3 PUFAs mediate their effects.

The cytoskeleton is an extensive framework of protein fibers in the cytoplasm. There are three main types of cytoskeletal polymers: actin filaments, microtubules and intermediate filaments which influence various mechanical and chemical stimuli within and between cells as well as subcellular structures [12, 13]. Changes of length, concentration and attachments to each other or to the cell membrane of these filaments are essential processes for all biological functions [14]. Therefore, cytoskeleton dynamics affect almost every cell-biological process, such as growth, differentiation, division, and death, but are also responsible for cell shape and distribution, inter- and intracellular transport and signaling, motility and adhesion [15]. Several diseases such as cardiovascular and neurodegenerative diseases, the immunodeficiency syndrome, Wiskott - Aldrich syndrome (WAS), cancer or liver cirrhosis have now been associated with abnormalities in cytoskeleton proteins [16]. To better understand the effect of n-3 PUFAs on cytoskeletal assembly, we conducted an open-label intervention study with healthy middle-aged men to investigate the gene expression changes of cytoskeletal genes as well as the alterations in actin dynamics after n-3 PUFA supplementation.

## Results

### Subject characteristics and changes of lipid levels, fatty acid composition of red blood cell membranes and omega-3 index

All subjects completed the study and tolerated the treatment with fish oil capsules very well. Compliance of subjects was determined by the counting of remaining capsules as well as by the omega-3 index. The mean age of all participating subjects was 43.3 ± 4.4 years. After n-3 PUFA supplementation the triacylglycerol levels significantly decreased by 24.8% (Table 1). Furthermore, relative amounts of n-6 PUFA levels in the red blood cell membranes were reduced, while n-3 PUFA levels increased. In contrast, relative EPA and DHA levels increased, which is reflected by a significant increase of the omega-3 index by 47% (Table 1).

**Table 1:** **Anthropometric data, lipid levels and fatty acid content in red blood cell membranes measurements**

Parameters	t _0_	t _12_
Age [years]	43.33 ± 4.40	
Height [cm]	183.04 ± 1.32	
Weight [kg]	87.88 ± 10.60	89.25 ± 10.92
Body mass index [kg/m^2^]	26.17 ± 2.31	26.57 ± 2.37
Waist circumference [cm]	92.25 ± 8.31	93.67 ± 10.15
Hip circumference [cm]	102.17 ± 6.34	103.50 ± 7.30
**Lipid levels**		
Total cholesterol [mg/dl]	217.42 ± 59.14	226.33 ± 53.44
Triacylglycerol [mg/dl]	146.25 ± 63.37	109.92 ± 32.88^a)^
High-density lipoprotein cholesterol [mg/dl]	52.00 ± 11.35	54.33 ± 8.48
Low-density lipoprotein cholesterol [mg/dl]	144.83 ± 50.49	155.33 ± 46.37
LDL/HDL ratio	2.91 ± 1.09	2.94 ± 1.06
**Fatty acids**		
**Omega-6 series:**		
Dihomo-γ-linolenic acid (20:3 n6) [% of total fatty acids]	2.04 ± 0.56	1.44 ± 0.34^a)^
Arachidonic acid (20:4 n6) [% of total fatty acids]	15.62 ± 0.87	12.26 ± 1.46^a)^
Adrenic acid (22:4 n6) [% of total fatty acids]	3.21 ± 0.56	1.88 ± 0.35^a)^
Docosapentaenoic acid (22:5 n6) [% of total fatty acids]	0.72 ± 0.11	0.45 ± 0.08^a)^
**Omega-3 series:**		
Eicosapentaenoic acid (22:5 n3) [% of total fatty acids]	0.92 ± 0.21	3.76 ± 0.84^a)^
Docosapentaenoic acid (20:5 n3) [% of total fatty acids]	2.92 ± 0.20	3.67 ± 0.43^a)^
Docosahexaenoic acid (22:6 n3) [% of total fatty acids]	4.83 ± 1.20	7.15 ± 0.79^a)^
**Omega-3 index [% of total fatty acids]**	5.74 ± 1.37	10.91 ± 1.53^a)^

Results are presented as mean ± SD.

^a)^t_0_ values vs. t_12_ values of subjects were tested by paired student’s t-test; p < 0.05.

### Gene expression changes of cytoskeleton-associated genes after n-3 PUFA supplementation

**Figure 1:**
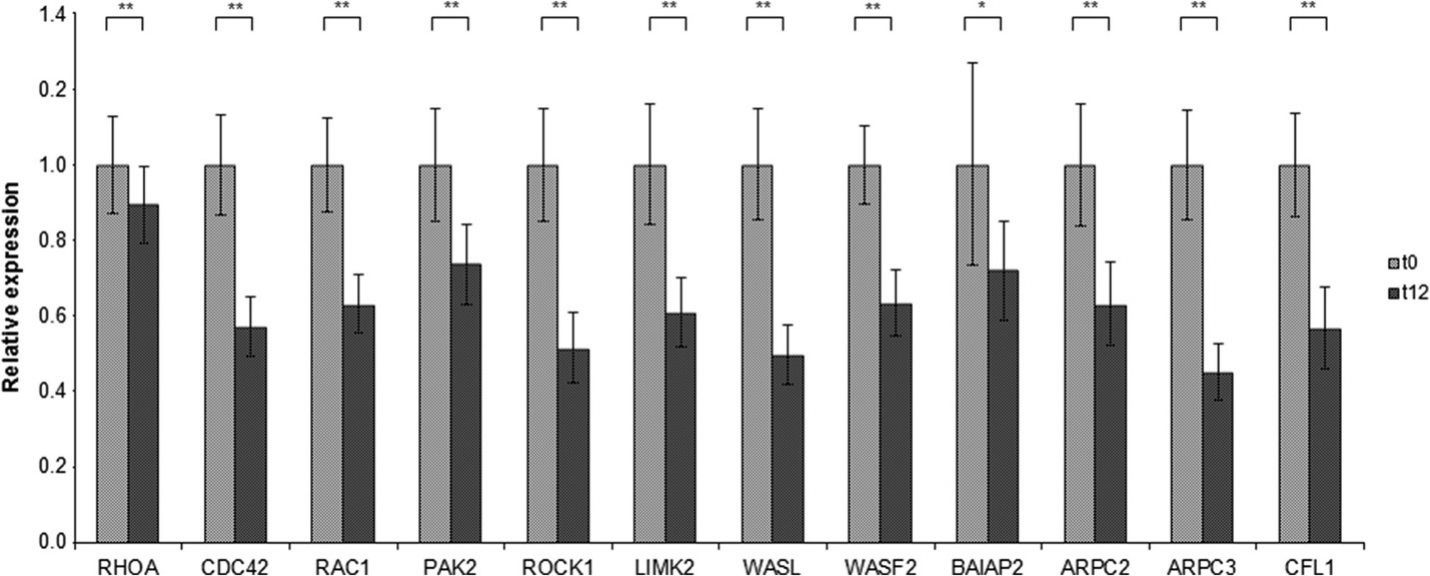
**Expression changes of cytoskeleton-associated target genes in healthy men after n-3 PUFA supplementation.** Transcript levels of RHOA, CDC42, RAC1, PAK2, ROCK1, LIMK2, WASL, WASF2, BAIAP2, ARPC2, ARPC3, and CFL1 were determined by qRT-PCR in healthy middle-aged men before (t_0_) and after 12 weeks (t_12_) of 2.7 g/d n-3 PUFA supplementation. Each sample was used in triplicate. Triplicates were averaged and corrected by two reference genes (GAPDH and RPS2). Corrected expressions of all subjects were compared with the baseline gene expression and the relative expression changes are displayed. Differences between time points were tested by Wilcoxon test. Asterisks mark the following significance levels: *p < 0.05; **p < 0.01.

The gene expression of three GTPases (ras-related C3 botulinum toxin substrate 1 (RAC1), ras homolog family member A (RHOA), cell division cycle 42RAC1 (CDC42)), three kinases (Rho-associated coiled-coil containing protein kinase 1 (ROCK1), p21 protein-activated kinase 2 (PAK2), LIM domain kinase 2 (LIMK2)), two Wiskott-Aldrich syndrome proteins (Wiskott-Aldrich syndrome-like (WASL), WAS protein family, member 2 (WASF2)), actin-related protein 2/3 complex (ARPC2, ARPC3), and cofilin (CFL1) were significantly down-regulated after n-3 PUFA supplementation (Figure 1). The strongest down-regulation was observed in WASL (-2.02) and ARPC3 (-2.21).

### Changes of F-actin content in red blood cells

**Figure 2:**
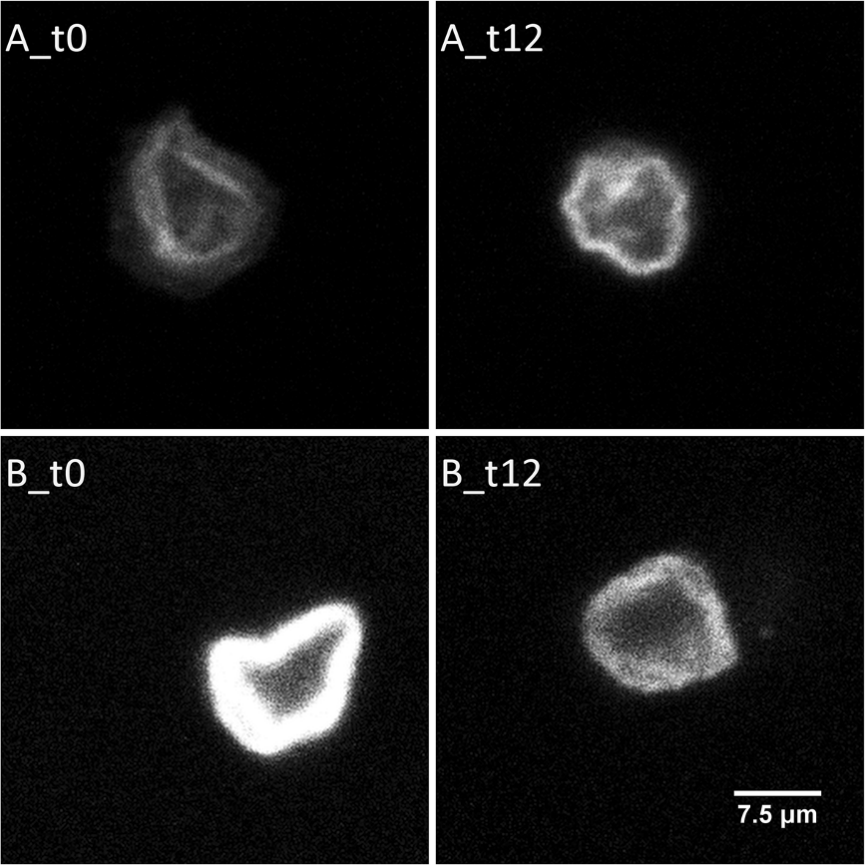
**N-3 PUFA induced changes in filamentous actin content in red blood cells.** Filamentous actin in red blood cells of healthy middle-aged men before (t_0_) and after 12 weeks (t_12_) of 2.7 g/d n-3 PUFA supplementation were labeled with phalloidin and whole cell fluorescence intensity was determined by confocal laser microscopy and data processing. The figure show examples for n-3 PUFA supplementation induced fluorescence intensity changes of two subjects. An increase **(A)** or a decrease **(B)** of phalloidin-labeled F-actin in red blood cells from baseline (t_0_) to study completion (t_12_) is shown.

The total fluorescence intensity of phalloidin-labelled F-actin in red blood cells was quantified at baseline and after twelve weeks of supplementation with n-3 PUFAs (Table 2). Of the twelve subjects, one half showed an increase and the other half a decrease in total fluorescence intensity, of which only three changes were significant. Whole group analysis revealed a significant decrease of phalloidin-labeled F-actin by 9.72% after n-3 PUFA supplementation. Figure 2 shows examples of decreased and increased fluorescence intensity after n-3 PUFA supplementation. Although no significant correlation between fatty acid composition and F-actin content could be investigated, a weak negative correlation (r = -0.397; p = 0.055) between the percentage DHA amount and F-actin content could be observed.

**Table 2:** **Fluorescence intensity of phalloidin-labeled filamentous actin in red blood cells**

	Fluorescence intensity of whole cell			
Subject	t _0_	t _12_	p-values ^a)^	Difference (t _12_ – t _0_ )
1	60.17 ± 18.78	65.68 ± 7.93	0.401	5.52
2	84.25 ± 4.92	37.91 ± 7.97	**< 0.001**	-46.35
3	52.12 ± 19.86	61.71 ± 11.37	0.252	9.58
4	39.93 ± 8.47	30.15 ± 8.03	0.054	-9.79
5	25.61 ± 8.35	37.76 ± 9.99	**0.014**	12.16
6	52.02 ± 13.51	64.95 ± 4.91	**0.028**	12.93
7	31.89 ± 8.27	23.47 ± 7.61	0.056	-8.42
8	36.11 ± 14.67	47.56 ± 21.59	0.263	11.45
9	42.87 ± 18.46	58.42 ± 15.92	**0.027**	15.55
10	67.53 ± 6.09	46.77 ± 19.22	**0.020**	-20.77
11	66.88 ± 9.87	62.84 ± 11.22	0.387	-4.05
12	59.17 ± 5.32	21.24 ± 11.67	**< 0.001**	-37.94
	**t**_**0**_	**t**_**12**_		
**Group average** ^**b)**^	51.55 ± 20.28	46.54 ± 19.67	**0.040**	-5.01

Ten images were taken of each subject sample (n = 12) at each time point. Therefore, a total of 240 images (120 cells at each time point (t_0_ and t_12_)) were analyzed by Fiji software package [30]. Firstly, all images were calibrated for intensity ranges. Secondly, all images were filtered by a 2 μm median filter. An optimal threshold was selected by multiple testing and applied to all images (selected threshold: Li). After this post-processing, the overall fluorescence intensity of phalloidin-labeled filamentous actin in each cell was measured. Results are presented as mean ± SD. Statistically significant differences are marked in bold.

^a)^t_0_ values vs. t_12_ values of subjects were tested by paired student’s t-test.

^b)^n = 120.

A bivariate Pearson’s correlation analysis between baseline fluorescence intensity and changes of intensity (Δt_12_ - t_0_) revealed that the higher the baseline intensity was, the stronger F-actin decreased during intervention. The correlation coefficient was r = -0.641 (p < 0.05).

## Discussion

Incorporation of n-3 PUFA in cell membranes affects a wide spectrum of cellular activities, including the actin cytoskeleton [17]. The influence of n-3 PUFA on cytoskeleton dynamics may prove a novel pathway for n-3 PUFA actions. The actin cytoskeleton plays an important role in various processes accompanied with the organization, formation and connection of cellular structures [13, 18]. Thus, changes of the regulation or function of cytoskeletal organization can contribute to diseases such as neurological disorders, CVD, WAS, or cancer [18]. In a previous whole genome gene expression study with humans [9–11], we found an altered regulation of the expression of several cytoskeleton-associated genes [GSE34898] in response to n-3 PUFAs treatment. In order to discover the possible influence of n-3 PUFAs on cytoskeleton assembly, we conducted another intervention study with healthy middle-aged men and investigated the effects of n-3 PUFA supplementation on the expression of related genes as well as alterations in actin dynamics. To the best of our knowledge, this is the first human study analyzing n-3 PUFA-induced gene expression changes as well as the differences of F-actin content in red blood cells.

Gene expression analysis of cytoskeleton-associated genes revealed a down-regulation of GTPases (RAC1, RHOA, CDC42), kinases (ROCK1, PAK1, LIMK), Wiskott-Aldrich syndrome proteins (WASL, WASF2), actin-related protein 2/3 complex (ARPC2, ARPC3), and cofilin 1 (CFL1) after n-3 PUFA supplementation (Figure 1), suggesting that n-3 PUFAs influence the actin cytoskeleton in healthy men.

Comparable data of gene expression changes are available only from a limited number of animal [19–22] and *in vitro* studies ([23, 24] abstract only). In these studies, n-3 PUFA supplementation resulted in an up-regulation of actin-related protein-2 in rat brains [19] as well as WASL in the precentral gyrus of the cerebral cortex of baboon neonates after a low (0.33 w/w% DHA) or high (1.00 w/w% DHA) PUFA diet for 12 weeks [21], respectively. In contrast, incubation of prostate cancer cells with EPA and DHA reduced the protein expression of GTPases (rac1, rac2 and cdc42) [24] (abstract only)].

In addition to these results, in this study immunofluorescence analysis of phalloidin-labeled F-actin in red blood cells showed a significant F-actin reduction of 9.72% but only for group evaluation (Table 2). In fact, variability in F-actin content between subjects was high and showed little marked direction. Hence, these data do not convey a clear picture impeding a generalized conclusion. Probably, baseline levels of intensity might have a relevant influence on F-actin changes. Here, we have seen more pronounced changes of immunofluorescence intensity when the baseline F-actin in red blood cells was higher. On individual level, no clear differences in the baseline characteristics of the subjects were observed. It should also be noted that high variations results from interindividual but also analytical variability.

A reduction of F-actin content in red blood cells has also been shown after n-3 PUFA incubation (50 μM for 48 h) of human trabecular meshwork cells which resulted in changes of the F-actin cytoskeleton, in particular, prevention of oxidative stress-induced (1 h 300 μM hydrogen peroxide) stress fibers and cross-linked actin network formation [25]. In addition, n-3 ethyl ester incubation in murine heart endothelial cells leads to a reduced migration of cells into induced wounds as well as a changed assembly of actin filament and focal adhesion in migrating and non-contacting cells [26]. Sakamoto and co-workers determined increased F-actin protein expression in the hippocampus of adult mice by 60% after supplementation with 300 mg/kg DHA for four weeks [27].

However, an increase or decrease of F-actin does not necessarily imply an advance or decline in function. Rather, an architectural and ultra-structural reorganization is to be expected.

Expression changes of cytoskeletal genes as well as changed F-actin content investigated in animal and *in vitro* studies are only partially transferable to the expression and F-actin changes observed in the current study, which are mainly attributable to analyzing different species and compartments. The results of the expression changes of cytoskeleton-associated genes and altered F-actin content of red blood cells after n-3 PUFA supplementation in this study are inconsistent. We cannot clearly indicate that n-3 PUFAs influence the actin cytoskeleton assembly by the regulation of cytoskeletal gene expression in healthy subjects. One can speculate that the organization of the actin cytoskeleton might be induced by n-3 PUFA supplementation, which consequently modifies cellular signaling [28]. Further studies are required to clarify mechanisms by which n-3 PUFA influence membrane micro-organization and modulate biological responses. Additionally, other polymers and actin isoforms which might be linked to disease and cytoskeletal organization as well as degeneration must be taken into account.

The study has a number of potential limitations. Although, n-3 PUFAs appear to regulate cytoskeleton-associated gene expression and change F-actin content in red blood cells, it is uncertain whether this would result in cellular consequences. This is mainly due to the fact that cytoskeleton markers, especially Rho-GTPases, are tightly regulated and post-translational modified, which finally determine their localization and function. Therefore, further studies analyzing cytoskeleton markers on protein level and cellular processes (i.e. cell division) in model systems are needed to verify the results and transfer it to cellular consequences.

## Conclusions

In conclusion, n-3 PUFA supplementation induced expression changes of cytoskeleton-associated genes on a transcriptional level in blood cells indicating that n-3 PUFAs influence the actin cytoskeleton. However, the interindividual variance in the F-actin content in red blood cells was high, which might mask effects of n-3 PUFA.

## Methods

This controlled explorative intervention study was conducted at the Institute of Food Science and Human Nutrition, Leibniz University of Hannover, Germany, and performed with respect to Good Clinical Practice Guidelines. The approval of the Freiburg Ethics Commission International (feki Code: 012/1707) was received.

### Subjects

Twelve healthy men aged between 36 and 49 years were recruited. The suitability of volunteers was checked by determination of blood parameters and by an admission questionnaire on diet, lifestyle and diseases. Exclusion criteria were defined as: smoking; body mass index > 35 kg/m^2^; chronic cardiovascular or liver diseases; gastrointestinal disorders; blood coagulation disorders and intake of coagulation-inhibiting drugs; renal failure; intake of any corticosteroids, lipid-lowering or anti-inflammatory drugs; periodic intake of laxatives; regular use of dietary supplements containing n-3 PUFA, phytosterols, polyglucosamines, and other lipid-binding ingredients or daily eating of fatty fish; allergy to fish or fish oil; and participation in another clinical study < 30 days before the study start or at the same time. Written informed consent was obtained from all participants.

### Study design

Subjects ingested six fish oil capsules per day for a period of twelve weeks. The daily intake of n-3 PUFA was 2.7 g (1.56 g EPA and 1.14 g DHA). The subjects were instructed to take three capsules in the morning and three in the evening together with food and a glass of water. Usual exercise and dietary habits should be maintained throughout the intervention time. Additionally, participants completed a questionnaire to obtain information about changes in medication, diet (e.g. changes in weekly fish intake, preferred fish dishes or species, respectively) and lifestyle habits (e.g. physical activity), as well as tolerability to the capsules.

### Determination of fasting serum lipids

Fasting venous blood samples were collected in BD Vacutainer® Blood Collection Tubes (Becton Dickinson, Heidelberg, Germany) at baseline and after twelve weeks of supplementation. The plasma lipid levels were determined by specific enzymatic color reactions from an external contract laboratory (LADR, Hannover, Germany). The results are presented as mean ± SD (Table 1).

### Determination of red blood cell membrane fatty acid composition

In order to determine the relative fatty acid composition in red blood cell membranes, samples were centrifuged at 2000 g for 10 min. Red blood cell concentrate was transferred to a new tube and stored at -80°C until analysis. Red blood cell membrane fatty acid composition including the omega-3 index, given as EPA and DHA, was analyzed at baseline and after twelve weeks of supplementation according to the omega-3 index methodology [29]. Results are presented as mean percentage of the total fatty acids ± SD (Table 1). The coefficient of variation for EPA and DHA was 5%. Quality was assured according to DIN ISO 15189.

### Determination of F-actin content in red blood cells by confocal laser scanning fluorescence microscopy

In order to obtain immunofluorescence imaging, the blood samples were centrifuged at 1200 g for 5 min, red blood cell concentrate was transferred to a new tube and mixed with equal amounts of 100% glycerol. Samples were frozen and stored at -80°C following the high-glycerol/slow-freeze method until analysis. Red blood cell concentrate-glycerol mixture was thawed at 37°C in a water bath. An amount of 10 μl of thawed sample was transferred to adhesion slides (Dianova GmbH, Hamburg, Germany). A second slide was arranged at an angle of 30° to disperse the blood over the slide’s length. The blood film was incubated for 30 min in a wet chamber to facilitate the adhesion of red blood cells to the slides. After adhesion, the cells were fixed with 200 μl of 4% methanol-free formaldehyde (Thermo Scientific, Rockford, USA) within 15 min. Permeabilization and staining were performed in one step using 100 μl of staining buffer containing 1xPBS, 0.1% Triton-X (Sigma-Aldrich, Hamburg, Germany) and 5 μl Alexa Fluor 488 Phalloidin (New England Biolabs GmbH, Frankfurt, Germany). The slides were incubated for 30 min in a dark wet chamber. Subsequently, the slide was dried by gentle patting on an absorbent cloth. An amount of 10 μl of ProLong Gold Antifade reagent (New England Biolabs GmbH, Frankfurt, Germany) was pipetted onto the slide and covered by an ethanol-cleaned cover slip to seal it. After a 20 min drying period in the dark, all sides of the slide were sealed with clear nail polish. The slides were stored in the dark at 4°C until microscopy.

Confocal laser scanning microscopy was performed with a Leica Upright DM-R connected to a TCS SP2 AOBS scan head. Alexa Fluor 488 excited at 495 nm and was visualized between 500 and 545 nm with a band pass filter. Images were recorded as a matrix of 256 × 256 pixels. Ten images were taken of each sample with a 100× plan-apochromat 1.46 oil immersion objective (Nikon, numerical aperture 1.46). A total of 240 images (120 cells at each time point) were analyzed by the Fiji software package [30]. Firstly, all images were calibrated for intensity ranges. Secondly, all images were filtered by a 2 μm median filter. An optimal threshold was selected by multiple testing and applied to all images. After this post-processing, the overall intensity of phalloidin-labeled F-actin in each cell was measured. Results are presented as mean ± SD (Table 2).

### Gene expression analyses

Fasting venous blood samples were collected in PAXgene Blood RNA Tubes (PreAnalytiX, Hombrechtikon, Switzerland) at baseline and after twelve weeks of supplementation. Samples were incubated for 24 h in the PAXgene Blood RNA Tubes at room temperature and then stored at -20°C until RNA isolation.

The total RNA was isolated with the PAXgene Blood RNA Kit (PreAnalytiX, Hombrechtikon, Switzerland), according to the manufacturer’s recommended procedures. The RNA yield was quantified by a Nanodrop ND-1000 spectrophotometer (peQLab Biotechnologie GmbH, Erlangen, Germany) measurement. The quality of purified RNA was checked with an Agilent 2100 Bioanalyzer using RNA 6000 Nano Chips and a RNA 6000 Nano Kit (Agilent Technologies, Böblingen, Germany). Only RNA samples with an optical density 260/280 ratio of more than 1.8 were used for further analyses.

cDNA from each subject was synthesized twice using 1.0 μg of purified RNA and M-MLV reverse transcriptase (Promega, Mannheim, Germany), as well as random hexamer (Fermentas, St. Leon-Rot, Germany) and oligo(dT) primers (Carl Roth, Karlsruhe, Germany). Synthesized cDNA was diluted 1:12 with nuclease-free water and used for the quantitative real-time polymerase chain reaction (qRT-PCR) together with iQ SYBR Green Supermix (Bio-Rad Laboratories, Hercules, Ca, USA) and 5 pmol of both forward and reverse primers. The sequences for target and reference genes were retrieved from GenBank and the primers applied were manually designed with the Primer-BLAST tool of the National Centre for Biotechnology Information, which is based on the program Primer 3 [31]. The primer sequences used are listed in Table 3. Glyceraldehyde-3-phosphate dehydrogenase (GAPDH) and ribosomal protein S2 (RPS2) were identified as the most stable reference genes by the freely available algorithm geNorm version 3.5.

**Table 3:** **Nucleotide sequences of primers for quantitative real-time polymerase chain reaction**

	Gene symbol	RefSeq_ID	Sequence (5'-3')	
**Cytoskeleton-associated target genes**	RAC1	NM_006908.4	Forward	ACTGTCTTGCCAGATTACCGA
			Reverse	GAACCTCACAGACCCAAAGGA
	RHOA	NM_001664.2	Forward	TCCCGTTTTGTCACTTTTTCTGAT
			Reverse	TCAGGCGTGAAACCAATTCC
	CDC42	NM_001791.3	Forward	CAACATGGGGACCAGTCAGG
		NM_001039802.1		
		NM_044472.2		
			Reverse	TGGAAACTGCAACCAAAATAAGC
	ROCK1	NM_005406.2	Forward	CATGCAAGCGCAATTGGTAGA
			Reverse	GTCCAAAAGTTTAGCACGCA
	PAK2	NM_001128620.1	Forward	GCCTCACTCCACTGATTGCT
		NM_002576.4	Reverse	GGATCAAAGTCTTGAGGAGTGC
	LIMK2	NM_016733.2	Forward	CTGGCTGAGAACTTACGGACA
		NM_005569.3	Reverse	GAGCCCACCCGAGTATGAGT
	WASF2	NM_006990.2	Forward	AAGCTCCATGCTGAGACACC
			Reverse	GCGCCTTCAACGACCATTTT
	WASL	NM_003941.2	Forward	AATGTCTTCAGGGTAATGCCAA
			Reverse	CGAAGAATGGGTACTCTCTGC
	BAIAP2	NM_017450.2	Forward	TGTGGCACTCACTGCTTTTCTA
			Reverse	GGAAGTCGGATGCGAGATAACA
	ARPC2	NM_152862.1	Forward	GGTGAACAACCGCATCATCG
		NM_005731.2		
			Reverse	AGGACCCCATCGAAATCTGC
	ARPC3	NM_005719.2	Forward	CTGCCCCCAGAGAGACAAAA
			Reverse	CTTTCTCACCTTGGCTTTTGGA
	CFL1	NM_005507.2	Forward	CGGCTCCTACTAAACGGAAGG
		NM_00112720.6		
		NM_00112720.4		
		NM_002134.3	Reverse	GACACCATCAGAGACAGCCA
**Reference genes**	GAPDH	NM_002046.3	Forward	AAGGTGGTGAAGCAGGCGTCG
			Reverse	AATGCCAGCCCCAGCGTCAAAG
	RPS2	NM_002952.3	Forward	GCAACTTCGCCAAGGCCACCTT
			Reverse	TGGGTCTTGACGAGGTGGTCAGT

Relative expression ratios of genes, which were quantified by qRT-PCR, were calculated with the Gene Expression Macro tool of Bio-Rad, which is based on the algorithm of geNorm [32]. Firstly, normalization factors were calculated from the geometric mean of the reference genes GAPDH and RPS2. Furthermore, the baseline values were defined as control values so that relative expression values could be calculated. Therefore, the baseline samples are given a value of 1. The results are presented as relative expression values ± SE (Figure 1).

### Statistics

All datasets were tested for distribution and homogeneity of variance before statistical analysis. Differences between time points were tested by paired student’s t-test (normal distribution) or Wilcoxon test for datasets which were not normally distributed. Correlation coefficient was determined with Pearson’s correlation. In general, p-values ≤ 0.05 were interpreted as statistically significant. Statistical analysis of anthropometric and laboratory chemical data were processed with SPSS software version 21.0 (SPSS Inc., Chicago, IL, USA). Statistical analysis of expression and immunofluorescence data were analyzed using the statistical package R version 2.15.0.

## Electronic supplementary material

Additional file 1: **Brief summary of previous findings.** The two supplementary tables illustrate a selected number of regulated genes associated with cytoskeleton structure and/or function in normolipidemic **(Table S1)** and dyslipidemic subjects **(Table S2)**. (PDF 64 KB)

## Abbreviations

ARPC2: Actin-related protein 2/3 complex, subunit 2
ARPC3: Actin-related protein 2/3 complex, subunit 3
BAIAP2: BAI1-associated protein 2
CDC42: Cell division cycle 42
CFL1: Cofilin 1
DHA: Docosahexaenoic acid
EPA: Eicosapentaenoic acid
F-actin: Filamentous actin
GAPDH: Glyceraldehyde-3-phosphate dehydrogenase
LIMK2: LIM domain kinase 2
n-3: Omega-3
PAK2: p21 protein-activated kinase 2
qRT-PCR: Quantitative real-time polymerase chain reaction
RAC1: ras-related C3 botulinum toxin substrate 1
RHOA: ras homologue family member A
ROCK1: Rho-associated, coiled-coil containing protein kinase 1
RPS2: Ribosomal protein S2
t_0_: Baseline
t_12_: After twelve weeks
WAS: Wiskott-Aldrich Syndrome
WASF2: WAS protein family, member 2
WASL: Wiskott-Aldrich syndrome-like.
