# Supplementary material for: Effect of omega-3 polyunsaturated fatty acids on the cytoskeleton: an open-label intervention study
Source: Lipids Health Dis. 2015 Feb 14;14:4. doi: 10.1186/1476-511X-14-4 (PMC4429932; doi:10.1186/1476-511X-14-4)
Supplement: Supplementary file 1 — Additional file 1: Brief summary of previous findings. The two supplementary tables illustrate a selected number of regulated genes associated with cytoskeleton structure and/or function in normolipidemic (Table S1) and dyslipidemic subjects (Table S2). (PDF 64 KB) [file 12944_2014_1211_MOESM1_ESM.pdf]

Effect of omega-3 polyunsaturated fatty acids on the cytoskeleton:

an open-label intervention study

Simone Schmidt, Janina Willers, Sabine Riecker, Katharina Möller, Jan Philipp Schuchardt, Andreas Hahn

Additional file

2 Additional Tables (Table A1 and A2)

Additional file: Brief summary of previous findings

Table A1: Differential expressed genes in normolipidemic subjects after twelve week of supplementation with fish oil or placebo. Gene highlighted in red were up-regulated, genes highlighted in green were down-regulated, genes written in italics were regulated in both intervention groups (fish oil and placebo) with in opposite directions.

Intervention group	Gene name	Gen-ID	p-value
Fish oil	Beta-arrestin-1 (Arrestin beta-1)	408	< 0.01
	Myosin light chain kinase, smooth muscle (MLCK)	4638	< 0.05
	Wiskott-Aldrich syndrome protein family member 2 (WAVE-2)	10163	< 0.01
	Rho GTPase-activating protein 4	393	< 0.001
	GRIP1-associated protein 1 (GRASP-1)	56850	< 0.001
	Myosin-If (Myosin-Ie)	4542	< 0.01
	Centrosome and spindle pole-associated protein 1	79848	0.05
Placebo	Calmodulin (CaM)	808	< 0.01
	cofilin 1 (non-muscle)	1072	< 0.01
	Myosin-If (Myosin-Ie)	4542	< 0.05
	Centrosome and spindle pole-associated protein 1	79848	< 0.01

Table A2: Differential expressed genes in <u>dyslipidemic subjects</u> after twelve week of supplementation with fish oil or placebo. Gene highlighted in red were up-regulated, genes highlighted in green were down-regulated, genes written in italics were regulated in both intervention groups (fish oil and placebo) with in opposite directions.

Intervention group	Gene name	Gen-ID	p-value
Fish oil	Beta-2-syntrophin	6645	< 0.05
	Syndecan-3 (SYND3)	9672	< 0.05
	Testican-2 Precursor	9806	< 0.05
	Nischarin	11188	< 0.05
	stathmin-like 3	50861	< 0.05
	Myosin-7B	57644	< 0.05
	Myosin light chain 6B	140465	< 0.05
	Muscle, skeletal receptor tyrosine protein kinase Precursor	4593	< 0.05
	Myosin-IXb	4650	< 0.05
	Muskelin	4289	< 0.05
	Myosin regulatory light chain 2	4633	< 0.01
	Myosin light chain kinase, smooth muscle (MLCK)	4638	< 0.01
	Moesin (Membrane-organizing extension spike protein)	4478	< 0.05
	Myosin-VIIb	4648	< 0.05
	Supervillin	6840	< 0.05
	Dynein light chain 1, axonemal	83544	< 0.05
	Syndecan-3 (SYND3)	9672	< 0.05
	Integrin alpha-3 Precursor	3675	< 0.05
	Triple functional domain protein	7204	< 0.05
	Cadherin-5 Precursor	1003	< 0.001
	Kinetochore-associated protein NSL1 homolog	25936	< 0.001
	Ankyrin repeat domain-containing protein 46	157567	< 0.01

	Dedicator of cytokinesis protein 9	23348	< 0.05
	Protein phosphatase 1 regulatory subunit 12B	4660	< 0.05
	Keratinocyte growth factor Precursor	2252	< 0.01
	Tensin-1	7145	< 0.01
	Myomegalin	9659	< 0.001
	Agrin Precursor	375790	< 0.05
	Transcriptional enhancer factor TEF-3	7004	< 0.05
	A-kinase anchor protein 13	11214	< 0.05
	Serine/threonine-protein kinase MRCK beta	9578	< 0.05
	F-actin-capping protein subunit beta (CapZ beta)	832	< 0.01
	Beta-arrestin-1	408	< 0.05
	Beta-arrestin-2	409	< 0.01
	Actin-like protein 7C	284382	< 0.001
	Rho GTPase-activating protein 25	9938	< 0.01
	Rho-related BTB domain-containing protein 3	22836	< 0.05
	Rho-related BTB domain-containing protein 2	23221	< 0.05
	Wiskott-Aldrich syndrome protein family member 3 (WAVE-3)	10810	< 0.05
	Cdc42 effector protein 5	148170	< 0.05
	Alpha-actinin-1	87	< 0.05
	Wiskott-Aldrich syndrome protein	7454	< 0.05
	Actin-related protein 2/3 complex subunit 3	10094	< 0.05
	Protein FAM65B	9750	< 0.01
	Protein Hook homolog 3 (hHK3)	84376	< 0.05
	Immunoglobulin-like and fibronectin type III domain-containing protein 1	91156	< 0.05
Placebo	Vimentin	7431	< 0.001
	BET1 homolog	10282	< 0.01
	Cyclic AMP-dependent transcription factor ATF-6 alpha	22926	< 0.001
	Putative myosin light chain kinase 3	91807	< 0.01

	PAS domain-containing protein 1	139135	< 0.001
	Wiskott-Aldrich syndrome protein (WASp)	7454	< 0.05
	Actin-related protein 2/3 complex subunit 3	10094	< 0.01
	Protein FAM65B	9750	< 0.001
	Protein Hook homolog 3 (hHK3)	84376	< 0.05
	Immunoglobulin-like and fibronectin type III domain-containing protein 1	91156	< 0.05
